# Optimizing the diagnostic power with gastric emptying scintigraphy at multiple time points

**DOI:** 10.1186/1471-2288-11-84

**Published:** 2011-05-31

**Authors:** Qingjiang Hou, Zhiyue Lin, Reginald Dusing, Byron J Gajewski, Richard W McCallum, Matthew S Mayo

**Affiliations:** 1 Department of Biostatistics, School of Medicine, University of Kansas Medical Center, Kansas City, Kansas 66160, USA; 2 Department of Medicine, Northwestern University Feinberg School of Medicine, Chicago, IL 60611, USA; 3 Department of Radiology, School of Medicine, University of Kansas Medical Center, Kansas City, Kansas 66160, USA; 4 School of Nursing, University of Kansas Medical Center, Kansas City, Kansas 66160, USA; 5 Department of Internal Medicine, Texas Tech University Health Science Center, El Paso, TX 79905, USA

## Abstract

**Background:**

Gastric Emptying Scintigraphy (GES) at intervals over 4 hours after a standardized radio-labeled meal is commonly regarded as the gold standard for diagnosing gastroparesis. The objectives of this study were: 1) to investigate the best time point and the best combination of multiple time points for diagnosing gastroparesis with repeated GES measures, and 2) to contrast and cross-validate Fisher's Linear Discriminant Analysis (LDA), a rank based Distribution Free (DF) approach, and the Classification And Regression Tree (CART) model.

**Methods:**

A total of 320 patients with GES measures at 1, 2, 3, and 4 hour (h) after a standard meal using a standardized method were retrospectively collected. Area under the Receiver Operating Characteristic (ROC) curve and the rate of false classification through jackknife cross-validation were used for model comparison.

**Results:**

Due to strong correlation and an abnormality in data distribution, no substantial improvement in diagnostic power was found with the best linear combination by LDA approach even with data transformation. With DF method, the linear combination of 4-h and 3-h increased the Area Under the Curve (AUC) and decreased the number of false classifications (0.87; 15.0%) over individual time points (0.83, 0.82; 15.6%, 25.3%, for 4-h and 3-h, respectively) at a higher sensitivity level (sensitivity = 0.9). The CART model using 4 hourly GES measurements along with patient's age was the most accurate diagnostic tool (AUC = 0.88, false classification = 13.8%). Patients having a 4-h gastric retention value >10% were 5 times more likely to have gastroparesis (179/207 = 86.5%) than those with ≤10% (18/113 = 15.9%).

**Conclusions:**

With a mixed group of patients either referred with suspected gastroparesis or investigated for other reasons, the CART model is more robust than the LDA and DF approaches, capable of accommodating covariate effects and can be generalized for cross institutional applications, but could be unstable if sample size is limited.

## Background

Gastric emptying scintigraphy at intervals over 4 h after a standardized meal is commonly regarded as the gold standard for measuring gastric emptying times. In practice, a simplified hourly measure of residual gastric contents is used for diagnosing gastroparesis defined as delayed Gastric Emptying (GE) in the absence of mechanical obstruction [1,2]. The technique measures radio-labeled food remaining in the stomach at hourly intervals after patient ingests a standardized meal [3] as an indicator of delayed GE. Because of differences in food used, techniques employed, and endpoints measured with GES, analysis and interpretation of the results vary among institutions [4]. Most previous studies have shown that delayed gastric emptying can be best indicated with gastric retention of >10% at 4- h after meal, which was established as the 95 percentile in gastric retention with normal volunteers through a multicenter study [5,6]. As always, a distribution-based diagnostic decision is arbitrary and may inevitably induce error because of misclassification. It is reported that the simplified approach has a specificity of 62% and a sensitivity of 93% [7]. Others use the percent retention at 2-h as the routine clinical measurements of GES [8], suggesting GES at 2-h or 3-h might be the best individual time point with higher diagnostic power. However, percent gastric retentions at different time points may also be subject to patient age and gender [5]. The lack of standardization in conducting GES along with differences in quantitative analysis used at various institutions may limit the clinical utility of the test, and presents problems if cross institutional data need to be evaluated. In 2008, on behalf of the American Neurogastroenterology and Motility Society and the Society of Nuclear Medicine, researchers from 13 US medical institutions jointly issued a consensus statement for standardization of GES across institutions [4]. In addition, issues requiring further investigation were identified by the consensus which include: 1) use of 3-h compared to 2- and 4-h results for detection of delayed GE; and 2) use of multiple time points (2- and 4-h) versus single 2- or 4-h values for further understanding of the clinical meaning of discordant results between 2- and 4-h scans.

Methodology on using different diagnostic markers to detect diseases or assess health related risks has been an active field of research [9-11]. With the rapid advances in genomic and proteomic technologies, the focus on biomarker-based disease detection and risk assessment has now shifted from a single biomarker to a panel of biomarkers since different markers may be sensitive to different aspects of a disease [12]. It is argued that, compared with a single clinical or genetic marker, a panel of multiple markers may contain a higher level of discriminatory information, particularly across large heterogeneous patient populations and for complex multistage diseases. This is important because using multiple markers simultaneously might lead to a new diagnostic measure with higher sensitivity [11].

When multiple markers are continuous and normally distributed, Fisher's LDA provides the best linear combination that maximizes the sensitivity over the entire specificity range uniformly under the multivariate normal distribution model [11]. When marker values deviate from normal, a Box-Cox power transformation can be used to improve the normal fit [13]. This approach has been found effective in estimating the AUC and ROC curves if the underlying true distributions, either on the original or on a transformed scale, are close to multivariate normal [11]. Otherwise, a rank based distribution free approach can be applied. The theoretical aspects of the best linear combination for biomarkers are well reported [[9,11,14-19], and [20]]. Fisher's LDA is easier to compute, while the DF approach is more robust to deviation from normal distribution, but is computationally prohibitive if more than two markers are involved [19]. We used the parameter estimates from LDA as a starting point for grid search with DF if marker space goes beyond bilateral. The CART model, on the other hand, provides another approach to optimize the diagnostic power when markers are multi-dimensional [21-23]. Rather than creating a new diagnostic measure like LDA or DF, CART uses a sequential process to identify the set of predictor variables, in their original scale, that best differentiate groups among the outcome variable of interest. It is particularly useful when there are many predictor variables that are highly correlated. It is computationally less intensive and easy to interpret, but can be unstable if the model is inadequately trained with limited data.

Most previous studies focus on deriving the best combination of markers that maximizes AUC for subjects whose disease status was known [11,14-20]. It is unclear if the diagnostic power for such a combination extends to the population with known marker values but unknown disease status or to the population whose marker values are not used for deriving the diagnostic parameters. With a standardized meal (see Materials and Methods) and the hourly GES technique (five 2-minutes imaging sessions) over 4 hour period, less camera time is required while results can be reproducible from hospital to hospital. This paper evaluates the statistical options that optimize the diagnostic power with GES measures at a few time points. Using the primary clinical diagnosis, defined by symptoms such as nausea, vomiting, early satiety, postprandial fullness, abdominal discomfort, and pain, in addition to evidence of delayed gastric emptying in the absence of mechanical gastric outlet obstruction, as the true status of gastroparesis, this study focuses on finding the parameters of the best linear combination of GES at different time points with training data, then, investigates and cross-validates its performance in test data that was not used for deriving the model.

## Methods

The Receiver Operating characteristic Curve (ROC) and the area under the curve in particular is a simple and meaningful measure to assess the usefulness of a diagnostic marker(s) [10]. Throughout this paper, ROC and AUC are used to compare across different methods and various models for the best diagnostic power of gastroparesis. The sum of false positive and false negative diagnoses over the entire sample was used to contrast the diagnostic power between LDA, DF and CART through jackknife (take one out) cross validation.

### Fisher's Linear Discriminant Approach (LDA)

Let *w*_*ij *_represents the *i*^*th *^marker value of the *j*^*th *^subject in the diseased group; and *v*_*ik *_be the *i*^*th *^marker value of the *k*^*th *^subject in the control group; where *i *= 1, ..., p; *j *= 1, ..., m; and *k *= 1, ..., n.

Suppose **X **and **Y **are vectors of marker values with multivariate normal distribution for the diseased and control groups respectively, then(1)

The coefficient vector, *α*_p × 1_, for the best linear combination of the combined marker vector from the diseased and control groups under the ROC criteria is:(2)

The AUC under the ROC curve is:(3)

Where, Φ denotes the standard cumulative normal distribution function.

The Specificity (F_a _(c)) and Sensitivity (H_a _(c)) are:(4)(5)

### Rank based Distribution Free approach (DF)

Under the rank based distribution free approach [19], the AUC can be calculated as the Mann-Whitney U statistic [24]:(6)

Note, for continuous distribution, Pr (*I *= 1/2) = 0.

Where, *w*_*ij*_is the *i*^th ^marker value for the *j*^th ^subject in the diseased group; *v*_*ik*_is the *i*^th ^marker value for the *k*^th ^subject in the control group; *i, j, k, m*, and *n *as defined in the previous section; *AUC*(*α*)is area under the curve by distribution free approach with optimal coefficient vector (α). Since ROC is invariant to monotone increasing transformation, the coefficient vector *α *in both LDA and DF approaches can be rescaled as (1, β_2/ _β_1_, β_3/ _β_1_, ... β_i/ _β_1_), where β_i/ _β_1 _represents the weight for the *i*^th ^marker value relative to marker 1 [19].

### Classification and Regression Trees (CART) model

Unlike LDA or DF, CART methodology identifies the set of predictors from all variables that best differentiate classes in the outcome of interest in a sequential process. At each step (node), CART algorithm first identifies the best predictor from all candidate variables and then searches through all values for the best predictor but uses only one cutoff point to divide the sample within the node into two sub-branches. It then searches through all predictor variables and identifies the best cutoff point from the best predictor within each sub-branch and repeats the process until a certain criteria, such as a minimum variance or a minimum group size, in all terminal nodes is met. At each node, CART seeks to classify the sample into groups such that maximum homogeneity of the child nodes is reached. When a terminal node is reached, the model gives the probability of belonging to a particular category for all remaining subjects that fall into that node. In practice, the minimum node size is usually set at 10% of the learning sample to avoid potentially over fitting the model such that the final decision tree is more likely to be useful for classifying populations with similar characteristics as that of the learning sample but was not used for deriving the CART model. In contrast to LDA approach, CART can be especially useful when the correlation patterns among predictor variables are not consistent over the entire range, because it is not necessary for the same variable to be optimal for all branch nodes of the entire tree [22].

### Parameter estimation

For LDA approach, we wrote a SAS/IML program [25] for calculating the mean (m), the variance (S) for diseased and control groups, and all parameter estimates are described in the method section. First, the coefficient vector *α*, AUC for the linear combination as well as for individual markers, was obtained with equations (2) and (3) from the training data. Then, the coefficient *α *was applied to the corresponding test data to obtain the linear combination score. Three threshold values corresponding to sensitivity levels at 0.7, 0.8, and 0.9 for the linear combination score were obtained with the gastroparesis data using  in equation (5). Each threshold value was used on the left out data to classify the case into either diseased or non-diseased group. The predicted status was then cross tabulated with the known disease category.

The DF approach started with an arbitrary starting point and then grid searched for the coefficients that maximize the Mann-Whitney U statistic with the training data in the following steps.

1) A linear combination score for each observation was obtained by multiplying the marker vector **X **with a starting coefficient vector, *α *_(1,*α*)._

2) The AUC corresponding to the coefficient vector *α*is calculated with equation (6).

3) Repeat the first two steps for every possible coefficient vector and aggregate the corresponding AUC and *α*.

4) Select the coefficient that maximized the AUC and identify the critical linear combination score value at 0.7, 0.8, and 0.9 sensitivity levels.

5) Applying the coefficient from step 4 to the test data to obtain the best linear combination score.

6) Use each critical value from step 4 in the test data to predict each case into either diseased or non-diseased group, then cross tabulate with the known disease category.

For CART model, we used the TREE package within R [26] environment for each of four hourly measures, and their combination along with patient age and gender to identify the best model. Cross-validation with a minimum size of 10 subjects for each terminal node was used to optimize the decision tree model using all observations [27]. AUC for each optimized decision tree was calculated to compare across all models. Then, one observation was taken out from the entire sample, and the remaining subjects used to build the decision tree, which in turn was used to predict the disease status of the left out observation. Performance for each decision tree was summarized with the total number of wrong predictions across the entire sample.

### Gastroparesis data

A total of 320 charts from patients aged 16 ~ 89 (42.8 ± 14.3 (mean ± std)), 255 (79%) female, with GES measures at 1 h, 2 h, 3 h and 4 h after a standard meal using a standardized method (5) were retrospectively collected at The University of Kansas Medical Center (KUMC). The study protocol was approved by the Institutional Review Board (IRB) at KUMC. During GES measurement, the fraction of meal consumed and the time taken for the consumption was recorded. Subjects with unusual percent meal consumed (e.g. <20% of the meal)/consumption time (e.g. >30 minutes) were excluded. All patients were either referred with suspected gastroparesis or investigated for other reasons because of self reported symptoms such as nausea, fullness, early satiety, vomiting, and bloating. Based on overall evaluation, in addition to hourly GES measures, the study physicians diagnosed 197 (62%) of the 320 patients with gastroparesis as the primary reason for above mentioned clinical symptoms and their hospital visits. Despite similar medical experiences, diseases other than gastroparesis were considered as the primary diagnoses for the remaining 123 patients. No significant difference in mean age (p = 0.12, by t-test) and gender (p = 0.99, by χ^2 ^test) were found between groups with and without gastroparesis. For each patient, gastric emptying scintigraphy was performed in the morning after an overnight fast with prokinetics stopped for at least 3 days. The standardized method for gastric emptying consists of the equivalent of two scrambled eggs (egg substitute ) labeled with ^99m ^Tc sulphur-colloid, 2 pieces of toast with jelly, and 4 oz of water with a total caloric value of 255 kcal. Anterior and posterior images of the stomach were taken immediately after eating, and then hourly for 4 hours [28].

## Results

**Figure 1:**
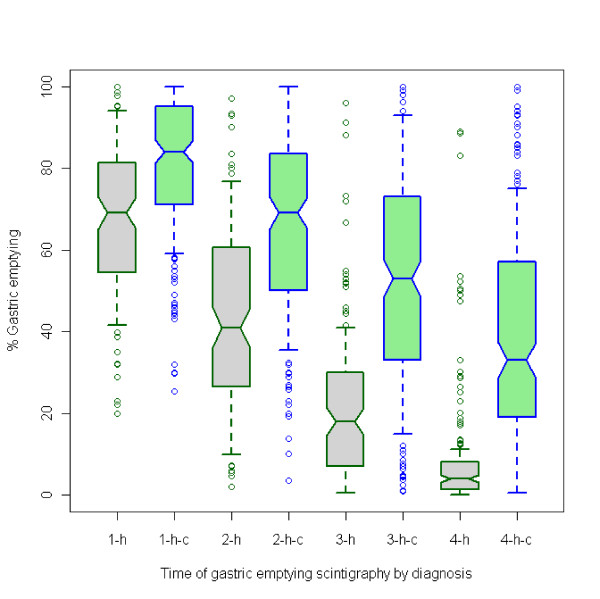
**Percent gastric retention at 1-h, 2-h, 3-h, and 4-h after meal for case (green) vs. control (grey).** 1-h-c, 2-h-c, 3-h-c, 4-h-c represents percent gastric emptying (GE) at 1-h, 2-h, 3-h, and 4-h after meal for patients diagnosed with gastroparesis; Whisker represents 70% interquartile range.

During repeated measurement of gastric emptying, percent retentions of the isotope in the stomach at 1-h, 2-h, 3-h, and 4-h after meal decreased with time and were highly correlated, especially for males and for patients with gastroparesis. Spearman correlation coefficient ranged from 0.34 (p < 0.001) between 1-h and 4-h for patients without gastroparesis to 0.93 (p < 0.001) between 3-h and 4-h for patients with gastroparesis. Overall, the distribution in percent retention deviated from normal, with the first two hourly values skewing toward the lower end, and the second two hours skewing toward the higher end (Figure 1).

### Diagnostic powers by LDA and DF approach

**Figure 2:**
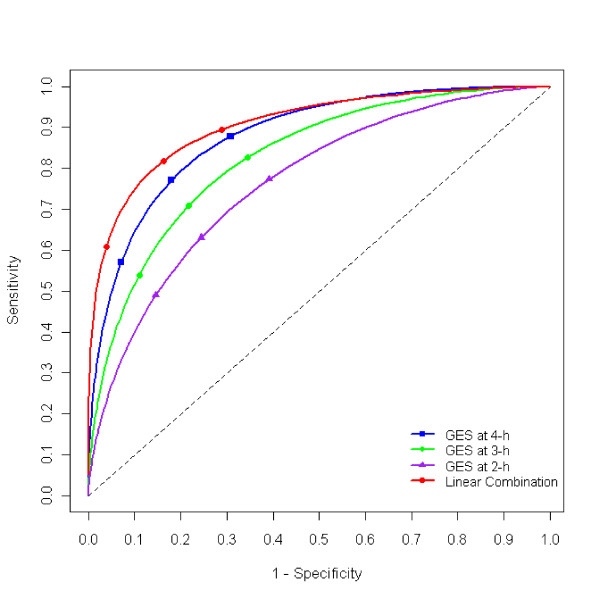
**Receiver's Operating Characteristic (ROC) Curve for hourly percent retention at 2-h, 3-h, 4-h, and their best linear combination**. Area Under the Curve (AUC) increased from 2-h to 4-h and maximized with the best linear combination of all three hourly measurements.

Hourly measures at 3-h and 4-h were previously reported as having the best diagnostic utility, we contrasted their best linear combination by both LDA (Figure 2) and DF approaches (Table 1). First, we estimated AUC for the two measurements along with the optimal coefficient for their best linear combination and the threshold values for the linear combination score at 0.7, 0.8, and 0.9 sensitivity levels by both LDA and DF approaches for all but 1 out of 320 subjects. The optimal coefficient, along with the three threshold values was then applied to the left out subject. By comparing the threshold values with the calculated linear combination score, the predicted gastroparetic status for the left out subject was recorded. The rates of false negative and false positive were obtained by repeating the jackknife process for all 320 subjects. Then, a Box-Cox power transformation was applied and the same analysis was repeated for the transformed data.

**Table 1:** Parameter estimates (Area Under the Curve (AUC) and coefficient for best linear combination) with Fisher's Linear Discriminant Analysis (LDA) and Distribution Free (DF) approaches

	4-h		3-h		Linear combination DF				Linear combination LDA			
	Raw	Tran	Raw	Tran	α	Raw	α	Tran	α	Raw	α	Tran
Mean	0.830	0.879	0.824	0.827	0.02	0.869	0.02	0.869	0.681	0.834	0.055	0.881
STD	0.002	0.001	0.002	0.002	0.00	0.001	0.00	0.001	0.032	0.002	0.002	0.001
Median	0.830	0.879	0.824	0.827	0.02	0.869	0.02	0.869	0.679	0.833	0.055	0.880
Range	0.013	0.009	0.011	0.009	0.00	0.008	0.00	0.008	0.284	0.012	0.017	0.009

Note: Raw stands for AUC for measurement in original scale;

Tran represents AUC for measurement in transformed scale;

α is coefficient for the best linear combination for measurement at 3-h after meal.

The best linear combinations of the two hourly measures increased the diagnostic power of its individual components by both approaches (Table 2), but the gain was limited, 3.9% for the DF, and 0.4% for the LDA approach. As expected, Box-Cox power transformation on individual markers improved the diagnostic power by the best linear combination with the LDA approach by 4.7% than that of the raw measures.

**Table 2:** False classifications (%) with jackknife cross-validation by Fisher's Linear Discriminant Analysis (LDA) and Distribution Free (DF) approaches

Criteria	3-h DF		4-h DF		Linear combination DF		Linear combination LDA	
								
0.7	24 (19.5%)	59 (30.0%)	16 (13.0%)	59 (30.0%)	15 (12.2%)	58 (29.4%)	15 (12.2%)	64 (32.3%)
0.8	37 (30.1%)	39 (19.8%)	21 (17.1%)	40 (20.3%)	21 (17.1%)	40 (20.3%)	20 (16.3%)	46 (23.4%)
0.9	60 (48.8%)	21 (10.7%)	27 (21.9%)	23 (11.7%)	28 (22.8%)	20 (10.2%)	27 (22.0%)	22 (11.2%)

Note: : model predicted disease status (1 for yes, 0 for no);

*y*: diagnosed disease status (1 for yes, 0 for no).

With the raw data, the differences in sum of false positive and false negative rates for the best linear combinations were 0.2%, 1.6%, and -6.5% between the DF and LDA approaches at the 0.7, 0.8, and 0.9 sensitivity levels. While the false negative rates by the LDA approach showed large deviation from that corresponding to the preset threshold levels (≤3.0%), those by the DF approach are relatively close (≤± 0.2%).

Data transformation improved the diagnostic power of the best linear combinations for both the LDA and DF approaches by decreasing the sum of false negative and false positive rates. The reductions are 2.2%, 2.8%, 13.8%, and -0.9%, -1.1%, 20.1% for the DF and LDA approaches, respectively. Interestingly, the largest improvement in diagnostic power is at the higher level of sensitivity for both approaches.

### Diagnostic powers with CART approach

Seven decision tree models, including the full model (4 hourly measurements along with the patient's age), the combinations of 2-h and 4-h, 3-h and 4-h, along with each hourly measures, were optimized through tree-pruning with minimum size for the final node of 10 subjects [26]. For all single time point models, percent retention at 4-h has the largest AUC of 0.865, followed by 3-h, 2-h, and 1-h, respectively (Table 3). The rate of false diagnosis by decision tree model with 4-h is 14.4% (28 out of 123 for patients without gastroparesis, and 18 out of 197 for those with gastroparesis), less than half of those who would be wrongly diagnosed by 1-h and 2-h points, and 37% ((73-46)/73) less than that -at 3-h. Including 2-h or 3-h along with 4-h with the decision tree did not increase the number of correct diagnoses over using 4-h alone as indicated by jackknife cross validation. These differ from results obtained from LDA and DF approaches, in which the linear combination of 3-h and 4-h showed slight improvement over using 4-h alone. However, decision tree model with either 4-h alone or its combination with 2-h or 3-h did not suffer in diagnostic utility compared with its counterpart models identified with either LDA or DF approach, regardless of data transformation. The CART model using all four hourly GES measures along with patient age was very interesting. For the criteria of gastric retention >10% at 4-h and <53% at 2-h, patients >47.5 years old were nearly 2 fold less likely to have gastroparesis (probability = 0.44) as those with age <47.5 (probability = 0.85).

**Table 3:** False classifications (%) with jackknife cross-validation by optimized Classification And Regression Tree (CART) models

Type of Misdiagnosis	1-h	2-h	3-h	4-h	2-h + 4-h	3-h + 4-h	4-h + 3-h + 2-h + 1-h + Age
	76 (61.8%)	28 (22.8%)	29 (23.6%)	28 (22.8%)	28 (22.8%)	28 (22.8%)	26 (21.1%)
	24 (12.2%)	69 (35.0%)	44 (22.3%)	18 (9.1%)	18 (9.1%)	18 (9.1%)	18 (9.1%)
Total Misdiagnosis	100 (31.3%)	97 (30.3%)	73 (22.8%)	46 (14.4%)	46 (14.4%)	46 (14.4%)	44 (13.8%)
AUC For Optimized Model	0.724	0.753	0.825	0.867	0.865	0.858	0.881

Note: : model predicted disease status (1 for yes, 0 for no);

*y*: diagnosed disease status (1 for yes, 0 for no).

## Discussion

Linear combinations of diagnostic markers obtained by LDA or DF approach usually lead to higher discriminate powers (larger AUC) than with its individual components. A simulation study (results not show here) indicated that the stronger the correlation among individual markers, the smaller the increase in AUC by their linear combination. The potential gain in diagnostic power, however, diminishes when the correlation between individual markers increase up to 0.7 and above. The effectiveness of using a linear combination decreases with the increase in magnitude of disproportion in covariance matrices between the diseased and control groups.

Small sample sizes lead to large variation in optimal coefficients for best linear combination and the corresponding AUC, especially for the LDA approach. As sample size increases, optimal coefficients and AUC by LDA or DF methods may or may not approach each other depending on the distribution of individual markers.

### Marker values normal

When the marker values are multivariate normal, the estimates by the LDA approach are very close to that of the DF method in terms of AUC, optimal coefficients, and the diagnostic power as indicated by simulation. With normal distribution and adequate sample size, the first two moments capture marker's location and scale parameter with small variation. In such cases, the LDA approach has the advantage in saving computation time (more than 100 fold less) without suffering in predictive power than the DF approach. Nevertheless, LDA cannot outperform DF as long as the searching grid for optimal coefficient with DF contains the point estimate by LDA. In other words, the limitation with DF is in computation, rather than methodology.

### Marker values not normal

When marker values deviate from normal distribution, the DF approach always leads to higher AUC for the best linear combination if the searching grid for optimal coefficients is fine enough. The downgrading performance with LDA approach is a direct result of using the means that is biased due to abnormality. Exponential distribution, for example, tends to have a long tail with a high degree of skewness, leading to a mean with a positive bias. As a consequence, the variances for markers are inflated and the AUC tends to be smaller. More importantly, the best linear combination obtained with LDA approach tends to overestimate the false negative rate and underestimate the false positive rate at the lower sensitivity level (Table 2), and do exactly the opposite at the higher sensitivity level. On the contrary, the best linear combination by DF approach is less affected by extreme values and tends to have higher diagnostic power while maintaining the preset sensitivity levels. This is important because, in practice, a fixed false negative rate represents a critical limit of tolerance in diagnostic medicine. Beyond such limit, the stake of loss-benefit ratio would increase, or, at least, the diagnostic decision is less cost effective.

### The effect of data transformation

Effective data transformation improves the normal fit and thus parameter estimation by LDA approach, but whether this improvement will hold in new data that is not included for parameter estimation remains unclear. Cross-validation with the gastroparesis data indicated that power transformation increased AUC and stabilized parameter estimates in the training sets, and, that such gains would translate into higher diagnostic power in the test sets (Table 2). Data transformation closed the gap in diagnostic power between the best linear combinations by LDA and DF approaches with the clinical data. Interestingly, the DF approach showed a consistent improvement with transformation across all levels of sensitivity at 0.7, 0.8, and 0.9. The trend with the LDA approach is not as clear. One reason might be the percent gastric retentions measured at 3-h and 4-h are so skewed that power transformation is not enough to put the measurements on nearly normal distribution.

### Optimizing the diagnostic power by GES measures with CART model

**Figure 3:**
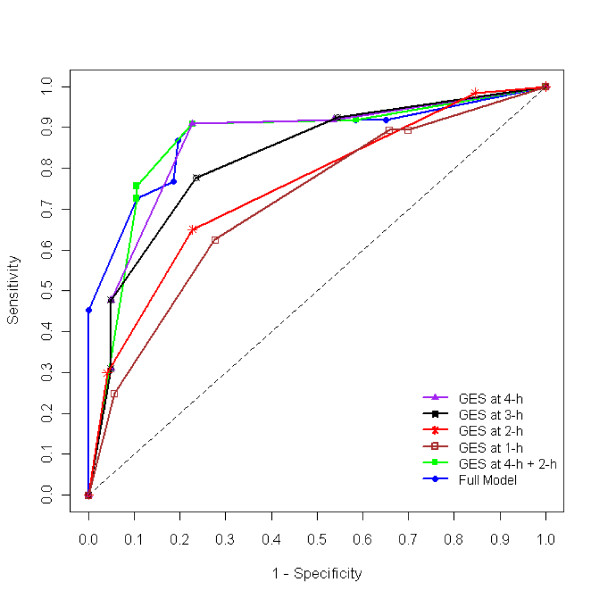
**Receiver's Operating Characteristic (ROC) Curve for classification tree models**. The full model has the largest area under the Receiver's Operating Characteristic Curve (ROC) followed by the combination of post-meal % retention at 2-h, and 4-h. Individually, the best time point is 4-h, followed by 3-h.

Unlike previous research with normal volunteers, our study population consisted with a mixed group of subjects either referred with suspected gastroparesis or investigated for other reasons. All subjects were experiencing some kind of gastric related symptoms but were not necessarily having gastroparesis as the primary reason. Consequently, gastric retention at 4-h for the control group (subjects with other primary diagnoses [non-gastroparesis]), in this study (10.3 ± 16.9%, mean ± std) were higher than that of the published with normal volunteers (5.4 ± 11.1%) (5). The control value in this study was primarily inflated by including more cases in the mild (10-15%), moderate (16-35%) and severe (>35%) categories as defined by a recently published reviews in the New England Journal of Medicine [7] and the American Journal of Gastroenterology [29]. We rely on the decision of clinical diagnosis by the study physician in reference to various symptoms and GES measures at different time points [30]. While using the inflated control value may reduce the sensitivity for gastroparesis, it will increase the chance to discover other major diseases that might be causing similar clinical symptoms. This is important because in real clinical settings, a lot of patients are experiencing complicated health problems with various comorbidities beyond or not explained purely by gastroparesis. Differentiating gastroparesis as a primary diagnosis would lead to different treatment approaches than recognizing it as comorbidity. The presence of patients with abnormal GES values in the control group, on the other hand, allows us to contrast the robustness of the three different models. Classification tree modeling is more appealing than the classic LDA and DF approaches in that: 1) it is computationally as efficient as the classic LDA approach; 2) it can handle any number of diagnostic markers and is robust to the presence of outliers and invariant to data transformation; and 3) it uses marker values in their original term and scale, thus easy to interpret in clinical practice. Figure 3 shows the optimized CART model with the highest diagnostic power for the gastroparesis data.

In practice, the critical value of 0.5 in probability can be used. That is, a patient is diagnosed with gastroparesis if, based on the post meal percent gastric retention measures along with covariates, the probability is greater than or equal to 0.5.

**Figure 4:**
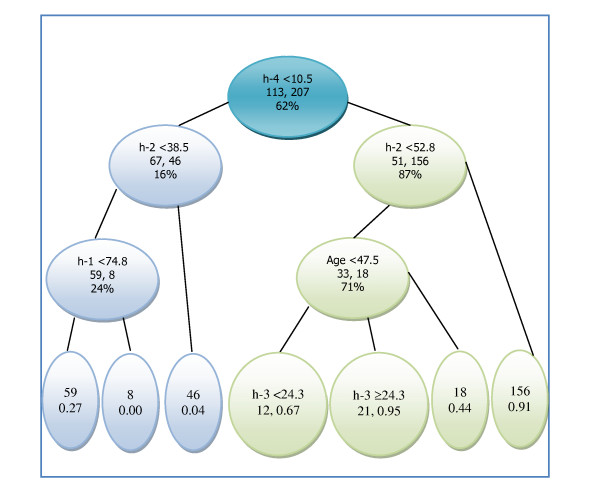
**The hierarchy of the Classification and Regression Tree (CART) model starts from the top with terminal nodes at the bottom**. Within each intermediate node are criteria for splitting, numbers of subjects, and percent of cases (diagnosed with gastroparesis) of all subjects within the node. Integers in the terminal nodes represent total number of subjects and decimal number at the bottom is the probability of a subject being diagnosed with gastroparesis.

The CART model was optimized from 4 hourly measures on percent gastric retention along with patient age and gender as covariates. Besides giving a probability estimate of having gastroparesis for patients falling into each node (Figure 4), the CART model uses the cut point of 10% gastric retention at 4-h as the main diagnosing tool with patients having gastric retention value >10%, more than 5 times likely to be gastroparesis (179/207 = 85.6%) than those with ≤10% (18/113 = 15.9%). This is consistent with the recommended >10% by the consensus report [4], despite the fact that the decision tree model derived the cutoff value from patient data while the consensus report established the cutoff point with normal subjects. Interestingly, the model also incorporated age into its predictor space. For the criteria of gastric retention >10% at 4-h and <53% at 2-h, hence normal at 2-h and abnormal at 4-h, we found that patients >47.5 years of age were nearly 2 fold less likely to have gastroparesis (probability = 0.44) as those with age <47.5 (probability = 0.85). Considering the large patient population used in this study, age could be an important covariate that helps diagnosing gastroparesis along with gastric retention at multiple time points. This is in agreement with a recent publication assessing gastric emptying in a large number of normal subjects utilizing the same gastric emptying methodology - the standardized egg meal over 4 hours. They found that older subjects had a faster gastric emptying rate than younger subjects who were slower [5]. A corresponding model with LDA approach would be problematic because 1) GE measures are not normally distributed; 2) gender is not a continuous variable; and 3) age effect is not consistent over the range of GES measures. With DF approach, GE data distribution is not a problem, but computation would be enormously difficult, even with the help of using a set of starting value that could be a rough estimate at best from the LDA approach. In addition, a model in multidimensional predictor space (6 in this case) with either LDA or DF approach is hard to conceive and might be meaningless to clinicians in practice.

## Conclusions

By contrasting CART, LDA, and DF approaches with the gastroparesis data, we hoped to be able to answer the two questions raised in the 2008 consensus statement [4]. In summary, we found that: 1) The diagnostic power of 4-h is higher compared to 2- and 3 -h results for detection of delayed GE, regardless of the method employed and whether the data is in original scale or in transformation; 2) Use of multiple time points (2-, 3-, and 4-h) will increase AUC and, as a result, the diagnostic power, versus single 2-, 3-, or 4-h values (Figure 2 &3). The improvement, however, is limited, especially with the LDA approach, because hourly measures are skewed and highly correlated. Therefore, we conclude that among LDA, DF, and CART, the CART model is more appealing because it is robust to outliers and invariant to data distribution, easy to compute, capable of using any number of predictor variables, and it offers easy interpretation. A potential downside with the CART model, however, lies in its limitation when the number of subjects (n) is small.

## Competing interests

The authors declare that they have no competing interests.

## Authors' contributions

QH conducted the literature review, developed the statistical analysis framework, and prepared the manuscript; ZL evaluated GES measurement, reviewed patients' charts, conducted data analysis, and actively involved in drafting the paper; BG and MM provided methodological guidance, supervised statistical analysis, and provided critical input to the manuscript. RD and RM supervised data collection, defined inclusion and exclusion criteria for the study cohort, interpreted the results and provided overall review of the research. All authors have read and approved the final manuscript.

## Pre-publication history

The pre-publication history for this paper can be accessed here:

http://www.biomedcentral.com/1471-2288/11/84/prepub
